# Editorial: Hospitals' Benefit to the Community: Research, Policy and Evaluation

**DOI:** 10.3389/fpubh.2020.00353

**Published:** 2020-08-05

**Authors:** Simone R. Singh, Tony Sinay, Penrose Jackson, Connie J. Evashwick

**Affiliations:** ^1^Department of Health Management and Policy, University of Michigan, Ann Arbor, MI, United States; ^2^Department of Health Care Administration, California State University, Long Beach, CA, United States; ^3^Vermont Public Health Institute, Burlington, VT, United States; ^4^Department of Health Services Management and Leadership, George Washington University, Washington, DC, United States

**Keywords:** community benefit, hospitals, non-profit hospitals, tax-exempt hospitals, IRS form 990 Schedule H, IRS form 990, hospitals and community engagement

In the United States (USA), “community benefit” (CB) encompasses the expectation, first written in 1969, that non-profit hospitals provide services to the communities they serve in exchange for tax-exempt status. Fifty years later, this Research Topic in *Frontiers in Public Health* collects a range of articles showing the current status of federal, as well as state, policies requiring hospitals to contribute to their communities in return for exemption from taxes.

USA non-profit hospitals have been required by the Internal Revenue Service (IRS) to report detailed information about their community benefit activities since 2007. In 2009, an Academy Health pre-conference, “Community Benefit: The Research Agenda for the First Five Years,” discussed the current state of non-profit hospitals' community benefit activities and offered a research agenda for the upcoming 5 years. Conference *Proceedings* (1) laid out critical issues to be examined as non-profit hospitals began reporting community benefit activities to the IRS as part of their annual tax return (IRS Form 990, specifically Schedule H). In 2010, the Patient Protection and Affordable Care Act (ACA) set forth further requirements for non-profit hospitals to maintain their tax-exempt status. These include the requirement that non-profit hospitals conduct a community health needs assessment (CHNA) every 3 years and develop an implementation strategy to address identified needs.

Now, more than 10 years after this inaugural conference and 50 years after the initial IRS ruling, detailed data on community benefit activities of non-profit hospitals and health systems are available to policymakers, researchers, and the public. CNHAs are ubiquitous and frequently involve not just hospitals but many organizations across the community. The activities outlined in hospitals' implementation strategies add numerous community-focused services to hospitals' portfolios. Evaluations analyze a wide variety of interventions, including health promotion and education programs, models of inter-entity collaboration, and the impact of social determinants of health on acute care. What do all these data, reports, and activities reveal? Have the research questions compiled in the 2009 Conference *Proceedings* been examined? What new information guides health policymakers and practitioners as they develop and implement policies related to non-profit hospitals' community benefit?

The 10 articles comprising the Research Topic shed light on the current status of non-profit hospitals' provision of community benefit. The 25 authors offer articles ranging from original research conducted on national samples of hospitals to personal perspectives. Twenty review editors and six associate editors contributed their own expertise in community benefit to the review process, enhancing the manuscripts. We thank all who contributed to this Research Topic in both formal and informal capacities.

Rozier analyzed 96 peer-reviewed articles pertaining to CB published over the past decade. He found a wide variety of studies, but no comprehensive analysis of the impact of the policies nor a singular focus of existing research and evaluation studies.

Evashwick and Jackson present a brief review of the history of CB regulations in the USA and point to the lack of specific theories or models on which to base public policy. They showcase the difficulty in conducting definitive evaluations of the impact of any CB activities when the assumptions about cause and effect relationships are imprecise and multiple entities in a community are involved in collaborative or independent interventions. They recommend each hospital specify its own logic model as a guide to desired outcomes and the basis for the corresponding evaluation.

Barnett adds to the history of the federal CB requirement, elaborating on how states approach it. He provides examples of recent changes by states, both following the federal example and taking independent, state-specific approaches. He advocates for a broad view of community organizations working together to improve health rather than depending upon a single entity.

Several studies focus on the CHNAs required by the ACA to be conducted by non-profit hospitals at least once every 3 years. Santos compiled a national sample to compare hospitals' CHNAs with their subsequent implementation strategies. She also examined collaboration between non-profit hospitals and local health departments in conducting and responding to the CHNAs.

Bias et al. contrast the role of individual community hospitals in conducting CHNAs with the role of the region-wide corporate health system. They conclude that although individual hospitals might have more detailed knowledge of their local communities, the corporate level health system can contribute in ways that enhance a strictly local focus. Both perspectives are beneficial.

Kaplan and Gourevitch describe lessons learned from a CHNA in New York. They provide examples of how the results supported creating several different community-oriented programs. They also describe how the CHNA's infrastructure formerly conducted by the hospital's planning department evolved to a collaborative effort involving a structured partnership with an array of community organizations which established the foundation for ongoing collaboration.

Ruggles highlights how a small hospital in rural Vermont served as a backbone institution for a multi-faceted collaborative community initiative. This case study raises the concept of “collective impact.” If a hospital works with other community organizations to improve health services or health status, can the hospital claim “community benefit” prowess, or do accomplishments belong to all the organizations? Reporting requirements have not been revised to reflect the value of collaborative efforts versus those of individual institutions.

Two articles bring the perspective of specialty hospitals. Carroll et al. consider how a rehabilitation hospital interacts with community agencies to address the multi-faceted needs of those with short-term and permanent disabilities. The hospital cannot meet all the needs of all its patients, but it can provide leadership to mobilize community services. Franz and Cronin explain how children's hospitals act differently than typical community hospitals because those they serve are likely to come from a much broader geographic area, encompassing multiple local communities, while focusing on one population segment—children and their families. To date, CB reporting and policy requirements have not been designed to recognize the differences that apply to specialty hospitals nor to acknowledge the regional impact noted by both Carroll et al. and Franz and Cronin. Similarly, “leadership” is difficult to quantify and report.

Turner et al. take an entirely different approach to CB. Rather than looking out, they examine how community benefit actions can contribute to internal operations. They explore how the move to value-based financing can utilize CB expectations to improve population health, thereby positioning the hospital to succeed financially under new payment systems.

Fifty years after the IRS handed its Regulatory Ruling about CB to the American Hospital Association and 10 years after the 2009 conference to set the research agenda, the reporting system has become more sophisticated and the evaluation metrics more complex. The range of subjects covered in this Research Topic shows the breadth and the complexity of the ways in which the formal CB policy has been implemented.

Historically, hospitals have contributed to their communities in ways that the institution and the community deemed appropriate. These articles beg the question, how do we move to better evaluate the impact of hospitals' efforts to improve community health? Clearly, we can cite the standard conclusion of ‘more empirical, data-driven research, evaluation, and policy analyses are needed’, especially now that national-level data on hospitals' CB are available and our ability to measure the impact of collaboration between hospitals and their community partners has advanced. Stimulating additional research and policy analysis on the CB activities of hospitals and their community partners will require more motivation and funding from stakeholders, including regulatory authorities, government agencies, and the private sector. The future may lie in hospitals of all types continuing to follow historic precedent to contribute to their communities not because of external regulation, but rather based on their mission and values and the increased recognition of the importance of creating healthy communities.

## Author Contributions

CE wrote the first draft of the article. PJ, SS, and TS edited the manuscript. All authors met together to discuss final revisions. All authors reviewed, added edits, and approved of the final version of the manuscript.

## Conflict of Interest

The authors declare that the research was conducted in the absence of any commercial or financial relationships that could be construed as a potential conflict of interest.
